# Synthesis and Kinetics of CO_2_-Responsive Gemini Surfactants

**DOI:** 10.3390/molecules29174166

**Published:** 2024-09-03

**Authors:** Yao Li, Xinyu Tang, Pujiang Yang, Yuhui Zhang, Jinhe Liu

**Affiliations:** College of Chemistry and Chemical Engineering, China University of Petroleum, Qingdao 266580, China; liyao172022@163.com (Y.L.); t764236216@163.com (X.T.); 19900020@upc.edu.cn (P.Y.); 19960026@upc.edu.cn (Y.Z.)

**Keywords:** CO_2_-responsive, Gemini surfactant, electrical conductivity, reaction dynamics

## Abstract

Surfactants are hailed as “industrial monosodium glutamate”, and are widely used as emulsifiers, demulsifiers, water treatment agents, etc., in the petroleum industry. However, due to the unidirectivity of conventional surfactants, the difficulty in demulsifying petroleum emulsions generated after emulsification with such surfactants increases sharply. Therefore, it is of great significance and application value to design and develop a novel switchable surfactant for oil exploitation. In this study, a CO_2_-switchable Gemini surfactant of *N*,*N*′-dimethyl-*N*,*N*′-didodecyl butylene diamine (DMDBA) was synthesized from 1, 4-dibromobutane, dodecylamine, formic acid, and formaldehyde. Then, the synthesized surfactant was structurally characterized by infrared (IR) spectroscopy, hydrogen nuclear magnetic resonance (^1^H NMR) spectroscopy, and electrospray ionization mass spectrometry (ESI-MS); the changes in conductivity and Zeta potential of DMDBA before and after CO_2_/N_2_ injection were also studied. The results show that DMDBA had a good CO_2_ response and cycle reversibility. The critical micelle concentration (CMC) of cationic surfactant obtained from DMDBA by injecting CO_2_ was 1.45 × 10^−4^ mol/L, the surface tension at CMC was 33.4 mN·m^−1^, and the contact angle with paraffin was less than 90°, indicating that it had a good surface activity and wettability. In addition, the kinetic law of the process of producing surfactant by injecting CO_2_ was studied, and it was found that the process was a second-order reaction. The influence of temperature and gas velocity on the reaction dynamics was explored. The calculated values from the equation were in good agreement with the measured values, with a correlation coefficient greater than 0.9950. The activation energy measured during the formation of surfactant was *Ea* = 91.16 kJ/mol.

## 1. Introduction

Surfactant systems usually only work at a certain stage, and when the stage is finished, they often need to be inactivated, thereby being well separated from the substance of value. For example, in oil extraction, easy demulsification is always expected after the oil is extracted in the form of emulsion using an aqueous solution of surfactant, so as to achieve effective separation between oil and water. In drug delivery, when the drug encapsulated in the surfactant self-assembly vesicles reaches the targeted diseased cells, the surfactant self-assembly structure needs to be destroyed, thus effectively releasing the drug. Regarding the regulation problem of surfactant systems, switchable surfactants have emerged. Switchable surfactants refer to a new type of surfactant that can be reversibly converted between “active” and “inactive” states under external stimulations such as temperature, electrochemistry, light, acid–base, and CO_2_ [1,2,3,4,5,6,7]. Among numerous switchable surfactants, CO_2_-switchable surfactants are widely preferred for their advantages such as low cost, environmental friendliness, simplicity, high efficiency, and reversibility [8,9,10].

Currently, the reported CO_2_-switchable surfactants mainly include the amidinyl system, the guanidine system, and the amine system [11]. In 2006, the Jessop research group [12] reported, for the first time in the Science journal, a CO_2_-switchable surfactant of long-chain acetamidine. Due to its unique mechanism of action and intelligent switching characteristics, once reported, it attracted wide attention in the academic community. Jiang et al. [13] found that the acetamidinium bicarbonate, which was formed by injecting CO_2_ into neutral *N*′-dodecyl-*N*,*N*-dimethylacetamidine, and SiO_2_ nanoparticles can form a stable milky liquid system through adsorption and wetting. The negatively charged silica particles can adsorb the amino cation through electrostatic interaction and become hydrophobic, forming a surfactant monolayer at the solid–water interface and stabilizing the n-octane-in-water Pickering emulsions. By injecting N_2_, the oil phase, surfactant, and nanoparticles were well separated, overcoming the difficulty that arises when demulsifying an emulsion in which conventional surfactants and solid particles act cooperatively. Since the guanidine group has a more stable conjugate action and a better affinity for protons than the amidine group, Qin et al. [14] designed and synthesized a dodecyl tetramethylguanidine compound (DTMG) and then studied the compound in terms of reversibility and foamability using phenolphthalein as an indicator. The color of the DTMG solution changed from colorless to red when phenolphthalein was encountered, and the red color gradually faded with the addition of CO_2_. Using the switch function of alkyl guanidine in controlling the foaming property, Chen et al. [15] applied alkyl guanidine in the foam-dyeing of circulating coatings. Scott et al. [16] systematically studied substances with amidine, guanidine, and tertiary amine as end groups, and pointed out that various nitrogen-containing end groups have different responses to CO_2_ due to their different pK_a_ values. Although the amidine surfactants show excellent performances, they are expensive [17]. Guanidine surfactants have a good affinity for protons, but they are deliquescent and require harsh conditions for easy deprotonation [18]. Therefore, the development and application of these two types of surfactants are restricted to a certain extent.

Tertiary amine surfactants are a low-cost option, and are usually easily deprotonated by injecting N_2_ at room temperature; they are reversible and have a higher efficiency. Using erucamide propyl dimethyl tertiary amine as the raw material, Zhang et al. [19] prepared a CO_2_-switchable wormlike micelle system, the viscoelasticity of which changed by five orders of magnitude when CO_2_ and air were injected alternately without heating. Using epoxy soybean oil as the raw material, Huang et al. [20] synthesized a CO_2_-switchable oligomeric surfactant, the viscosity of which in the form of a solution increased significantly with the injection of CO_2_, but it was restored to its initial low viscosity in the absence of CO_2_. Based on natural rigid rosin, Yan et al. [21] synthesized a CO_2_-switchable surfactant of MPANGG, which is a tertiary amine with two methyl groups and a side chain of PAHs. Both cationic and neutral MPANGG showed excellent surface activity, but cationic MPANGG exhibited a better emulsifying performance, which is mainly due to the electrostatic repulsion that occurs between the emulsified droplets.

At present, most CO_2_-switchable surfactants have a traditional single-chain structure with low surface activity. Therefore, the development of CO_2_-switchable Gemini surfactants with high surface activity is of great significance and application value. Most CO_2_-switchable Gemini surfactants are pseudo-Gemini surfactants that act through electrostatic interactions. Zhang et al. [22] mixed anionic surfactant SDS with *N*,*N*,*N*′,*N*′-tetra­methyl-1,3-propanediamine (TMPDA) with a 2:1 M ratio. Bubbling CO_2_ protonates TMPDA at two positions and can non-covalently bond to two SDS molecules, forming a pseudo-Gemini surfactant. These multi-component surfactants can shape wormlike micelles and, as a result, form a highly viscoelastic fluid. The removal of CO_2_ reversibly reshapes the wormlike micelles into spherical ones, reducing the viscosity significantly. Wang et al. [23] prepared a series of pseudo-Gemini surfactants by simply mixing long-chain fatty acids with polyetheramine at a molar ratio of 2:1, demonstrating their ability to stabilize CO_2_-responsive aqueous foams. Liu et al. [24] non-covalently linked different amines with oleic acid (OA) to form pseudo-Gemini surfactants in the form of amine-2OA. They found that the interfacial activity of the surfactants and the number of hydrogen bonds between the spacer and the oleic acid anion increased as the spacer length increased.

In this study, a novel Gemini CO_2_-switchable tertiary amine surfactant was developed, which includes an additional hydrophilic and hydrophobic group compared to traditional surfactants. The hydrophilic head group in the molecular structure is effectively connected with the binding groups through chemical bonds, so that the charges of the surface-active part are gathered. Furthermore, the two hydrophobic chains are arranged tightly due to the shortened distance, enabling more remarkable surface activity. At present, most research focuses on the synthesis methods and properties of switchable surfactants, but the quantitative characterization of the switching rate of surfactants has not been reported. For better use of the CO_2_-switchable surfactant, the surfactant was characterized with respect to its switching rate using a simple, non-destructive, and easily operated conductivity measurement method. The kinetic equation of the process for generating the surfactants with the injection of CO_2_ was studied, as well as the effects of temperature and injection rate of CO_2_ on the kinetics of the process for generating the surfactant. According to the kinetic equation, the amount of reaction under different external conditions can be quantitatively characterized; the extent of the reaction can be grasped, and the reaction conditions can be optimized theoretically. This work provides a basic theoretical foundation for its subsequent applications in industry.

## 2. Results and Discussions

### 2.1. Structural Characterization of DMDBA

**Figure 1:**
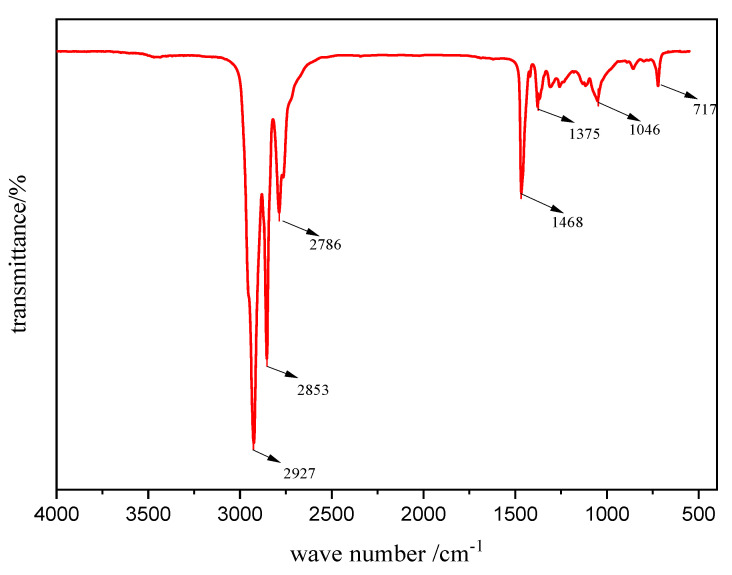
IR spectrum of DMDBA.

**Figure 2:**
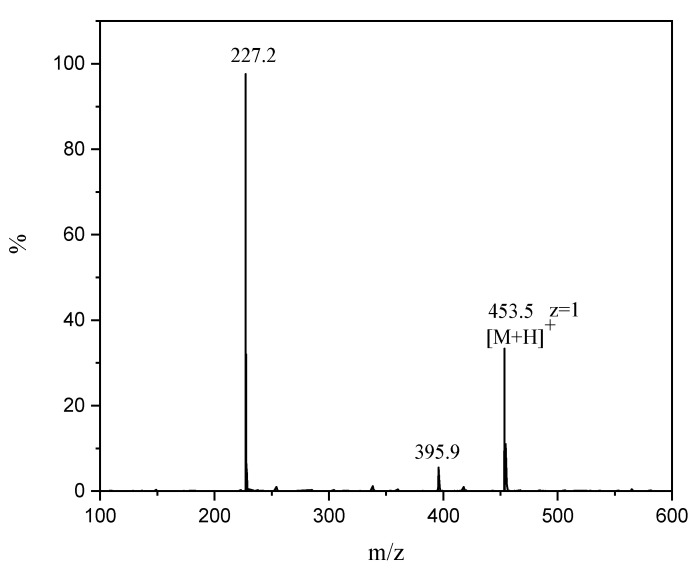
ESI-MS spectrum in positive ion mode of DMDBA.

**Figure 3:**
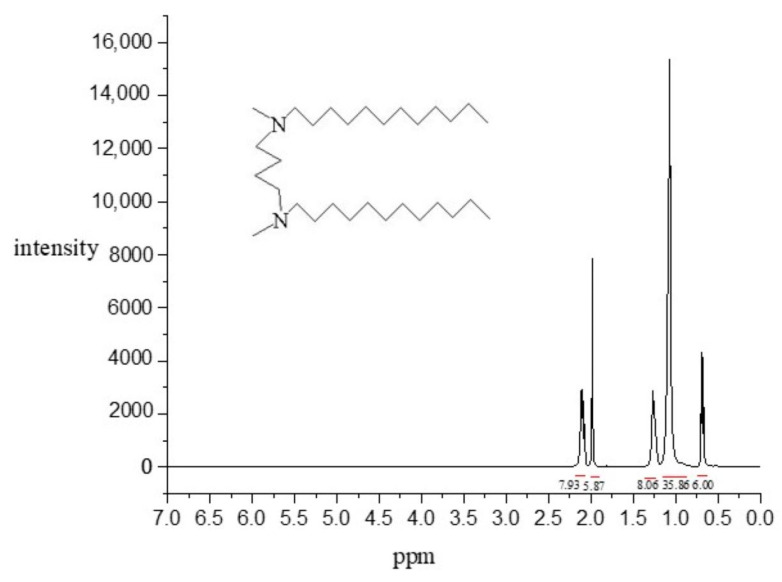
1H NMR spectrum of DMDBA.

Figure 1 shows the IR spectrum of DMDBA, in which the absorption peaks at 2927 cm^−1^, 2853 cm^−1^ and 2786 cm^−1^ are attributed to the C-H stretching vibrations of -CH_3_ and -CH_2_ groups; the absorption peak at 1468 cm^−1^ is attributed to the bending vibration of the -CH_2_ group; the absorption peaks at 1375 cm^−1^ and 1046 cm^−1^ are attributed to C-N stretching vibrations; and the absorption peak at 717 cm^−1^ is attributed to the out-of-plane bending vibration of C-H in the -(CH_2_)_n_ chain link. The IR absorption peaks of the sample are consistent with the characteristic functional groups of the target product, and the IR absorption peak due to the N-H bond is not observed within the wavenumber range of 3100–3500 cm^−1^, indicating that there is no residue of reactants in the synthesized product. Figure 2 shows the ESI-MS spectrum in positive ion mode, and the interpretation of this confirms that the mass–charge ratio of DMDBA is consistent with the theoretical one of 452.5. The ¹H NMR characterization results of Figure 3 are as follows: (400 MHz, CCl_3_, δ):0.69 (t, 6H, CH_3_C), 1.08 (m, 36H, C(CH_2_)_9_), 1.26 (m, 8H, -CH_2_CN-), 1.98 (s, 6H, -NCH_3_-), 2.10 (m, 8H, -NCH_2_-). The results of the ¹H NMR spectra are consistent with the hydrogen ratio of the target product in different chemical environments, and no other miscellaneous peaks are found in the spectra. Combining the results of the IR spectrum, MS spectrum, and ¹H NMR spectra, the synthesized compound was demonstrated to be the target product.

### 2.2. Switching Property of DMDBAH

**Figure 4:**
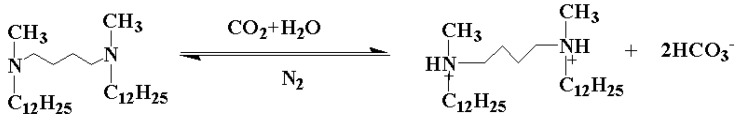
Reaction mechanism of switchable surfactant (DMDBA).

**Figure 5:**
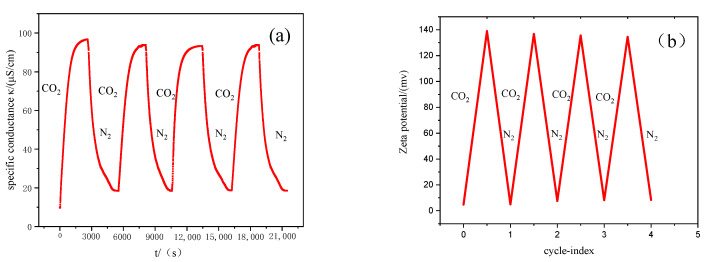
(**a**) Changes in conductivity after DMDBA was injected with CO_2_/N_2_; (**b**) changes in Zeta potential after DMDBA was injected with CO_2_/N_2._

The synthesized tertiary amine (DMDBA) is a neutral compound, and its solution contains almost no free cations or anions. However, after CO_2_ was introduced into the solution, the tertiary amine group was protonated into tertiary amine bicarbonate (DMDBAH) (Figure 4), which increased its solubility in water, thereby yielding a large number of negative and positive ions in the solution. Therefore, the conductivity of the solution increased rapidly, and the thickness of the double electric layer increased under the action of electrostatic repulsion, ultimately increasing the absolute value of the Zeta potential [25,26]. When the conductivity of the solution reached its maximum value, the solution was injected with N_2_ for deprotonation. Although not all values could completely return to the initial ones, the degree of reversibility was more than 90%, which would not affect the utilization of its CO_2_/N_2_-switchable response. When the same solution was repeatedly injected with CO_2_ and N_2_ four times, it showed a consistent pattern (Figure 5), indicating good CO_2_ responsiveness and cyclic reversibility.

### 2.3. Surface Properties of DMDBAH

**Figure 6:**
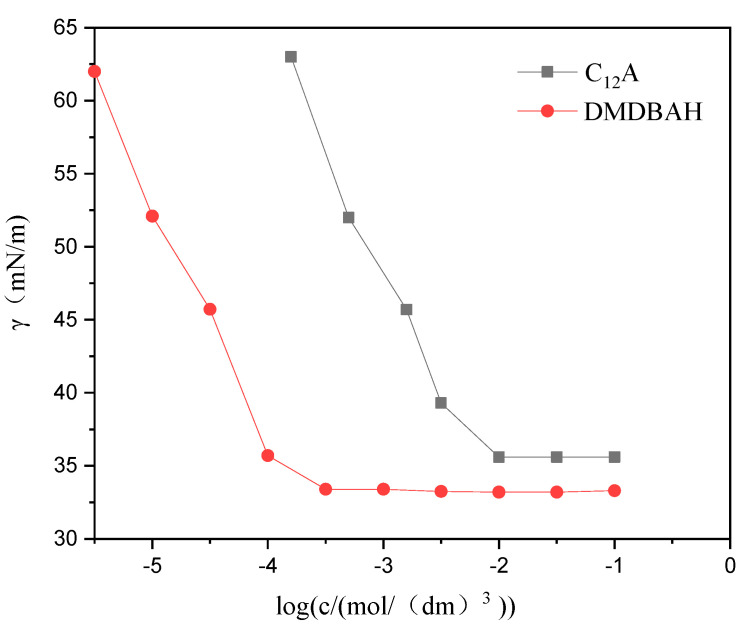
Surface tension curve of DMDBAH at 25 °C.

Figure 6 shows the surface tension curves of the cationic surfactants obtained by injecting CO_2_ into single-chain tertiary amine and the synthesized double-chain tertiary amine at different concentrations, respectively. It can be seen from the figure that the DMDBAH aqueous solution at low concentrations dramatically reduces surface tension, and with an increase in the concentration, the adsorption of surfactant molecules on the surface of the solution gradually reaches saturation. The decreasing trend of the surface tension of the solution slows down until an inflection point is reached, at which the minimum surface tension value is observed. Beyond this point, the surface tension experiences a plateau despite the continuous increase in concentration, showing the characteristics of typical surfactants [27]. As can be seen from Table 1, DMDBAH is an excellent surfactant with a lower CMC, higher surface adsorption efficiency (*p*C_20_), better aggregation efficiency at the air/water interface, and better ability to reduce water surface tension compared to those of the single-chain tertiary amine switchable surfactant C_12_A and other switchable surfactants with the same chain length [28,29]. This is mainly due to its special structure, which enables a close connection between two surfactant monomers, inhibits the separation between hydrophilic groups due to electrostatic repulsion, enhances the binding between hydrophobic alkane chains, and thereby makes aggregation into micelles easier and greatly improves the surface activity [30,31,32]. The smaller the CMC value, the lower the concentration required for the surfactant to form micelles in aqueous solution, and the lower the concentration needed to achieve surface saturation adsorption. This means that only a small amount of surfactant is required to play a role in wetting, emulsifying, etc., which can greatly save costs in industrial applications.

### 2.4. Wettability of DMDBAH

**Figure 7:**
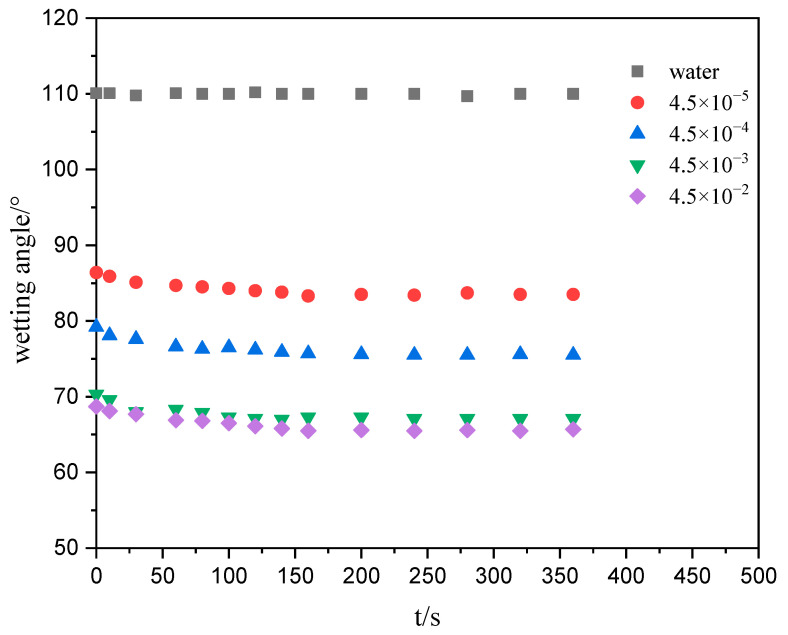
The contact angle between DMDBAH and paraffin wax varies with DMDBAH concentration and time at 25 °C.

Wettability can be determined by the contact angle of the surfactant solution on a hydrophobic film (paraffin film in this case). If the contact angle between the droplet and the paraffin wax is less than 90°, the surface is defined as highly wettable. It can be seen from Figure 7 that the contact angle on the paraffin film decreases with the increase in surfactant concentration and test time, which can be attributed to the increase in surfactant molecules at the interface. When the surfactant concentration exceeds 4.5 × 10^−5^ mol/L, the contact angle drops to below 90°, indicating that the surfactant has changed the wetting state of water on the surface of the paraffin film. When the surfactant concentration exceeds 4.5 × 10^−3^ mol/L, the contact angle stabilizes around 65° for a long time. With the increase in time, the contact angle no longer changes, indicating that the adsorption at the liquid–solid interface has reached saturation [33,34].

### 2.5. Emulsification/Demulsification Performance of DMDBAH

**Figure 8:**
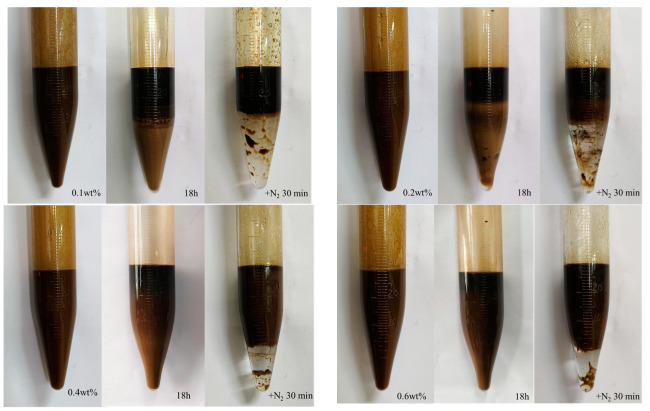
Dehydration situation of emulsions formed by different concentrations of surfactants and Gudao crude oil.

Table 2 shows the oil output of emulsions obtained with different concentrations of DMDBAH surfactant at different times. Under the same standing time, the smaller the volume of oil phase precipitated in the emulsion, the higher the stability of the emulsion. When the concentration of DMDBAH was 0.6 wt%, the least amount of oil phase was precipitated during the same period, and the emulsification effectiveness of the system was the best. This can be explained by the fact that more surfactant molecules are adsorbed on the liquid/liquid interface with an increase in surfactant concentration, which increases the strength of the interface film, thereby stabilizing the emulsion. When the surfactant concentration exceeded 0.4 wt%, the surfactant molecules were closely arranged at the interface and reached saturation. A further increase in the surfactant concentration has very little effect on the stability of the emulsion, and excessive surfactant would increase the difficulty of later demulsification. As can be seen from Figure 8, the emulsion formed from the crude oil from the Gudao oil field with different concentrations of surfactants was left at 25 °C for 18 hours. The stability of the emulsion decreased but without the production of water, and oil–water separation was achieved only if a demulsifier was added. However, with the injection of N_2_ into the emulsion, oil and water were separated at room temperature, which is simple, low-cost, pollution-free, and can be achieved under mild conditions. With the same N_2_ inlet time, the higher the DMDBAH concentration, and the lower the degree of oil–water separation.

### 2.6. Reaction Kinetics of DMDBA

**Figure 9:**
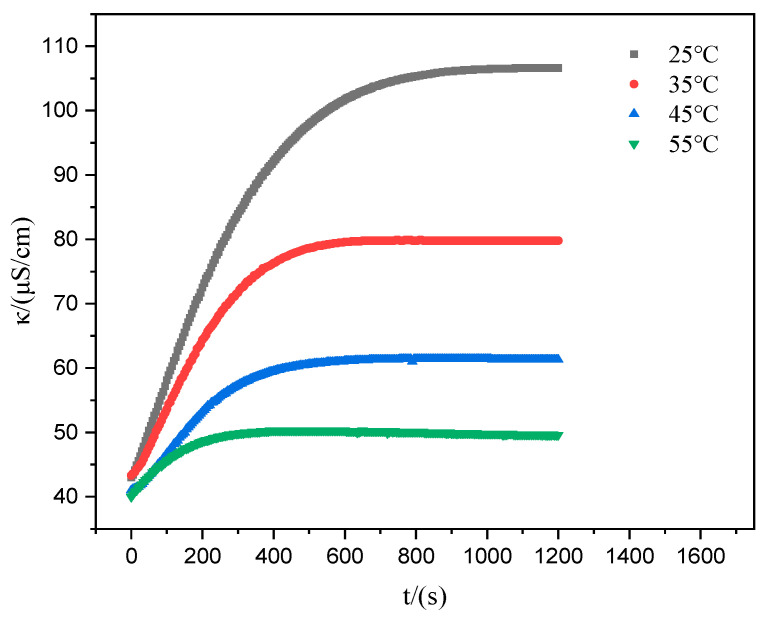
Changes in the conductivity of the surfactant formation process after injection of CO_2_ into DMDBA at different temperatures with time.

**Figure 10:**
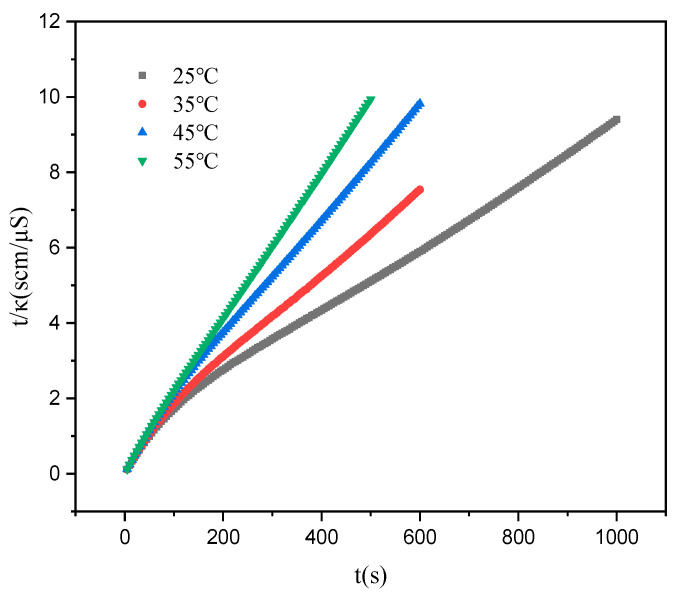
Kinetic fitting curve of the surfactant formation process after injection of CO_2_ into DMDBA at different temperatures.

DMDBA reacts with CO_2_ to generate the cationic surfactant of DMDBAH, which gradually increases the conductivity. When the reaction is complete, the conductivity remains dynamic and stable. Therefore, the rate of conductivity change could quantitatively characterize the reaction rate. The conductivity change during the formation of the surfactant with the injection of CO_2_ into the tertiary amine at different temperatures was fitted to the kinetic equation (Figure 9 and Figure 10). The correlation coefficients were all greater than 0.99 (Table 3), indicating that the formation process of surfactants at different temperatures followed the second-order kinetic law. The value of rate constant k increased with the increase in temperature and reached the maximum value at 55 °C, which indicates that the appropriate increase in temperature is conducive to accelerating the reaction. With the increase in temperature, the molecular motion rate increases, resulting in an increased probability of molecular collision per unit time, which then accelerates the bonding with CO_2_, i.e., a faster reaction rate. However, since the protonated tertiary amine bicarbonate is susceptible to decomposition by heat, increased temperature will decrease the degree of protonation (Figure 9) [28,35]. According to the Arrhenius formula ln*k* = −*Ea*/*RT* + lnA, the activation energy *Ea* = 91.16 kJ/mol can be obtained by the linear regression method from ln*k* and −1/*RT*.

**Figure 11:**
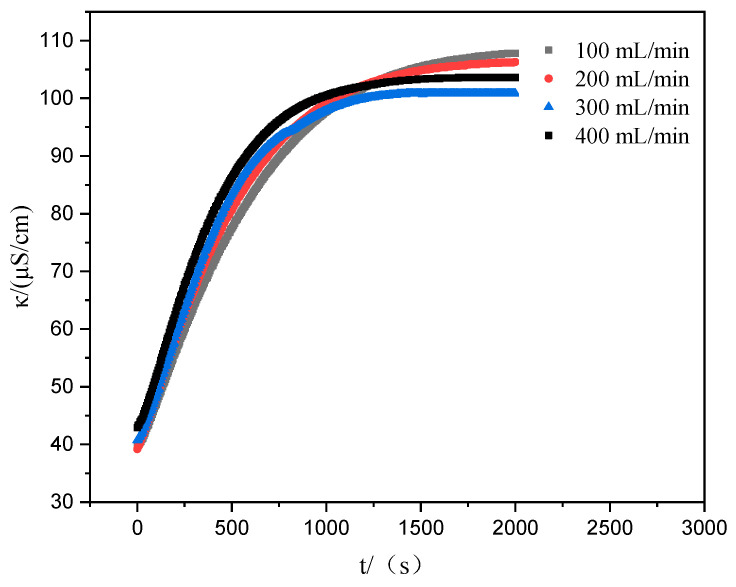
Changes in the conductivity of the surfactant formation process after injection of CO_2_ into DMDBA at different speeds with time.

**Figure 12:**
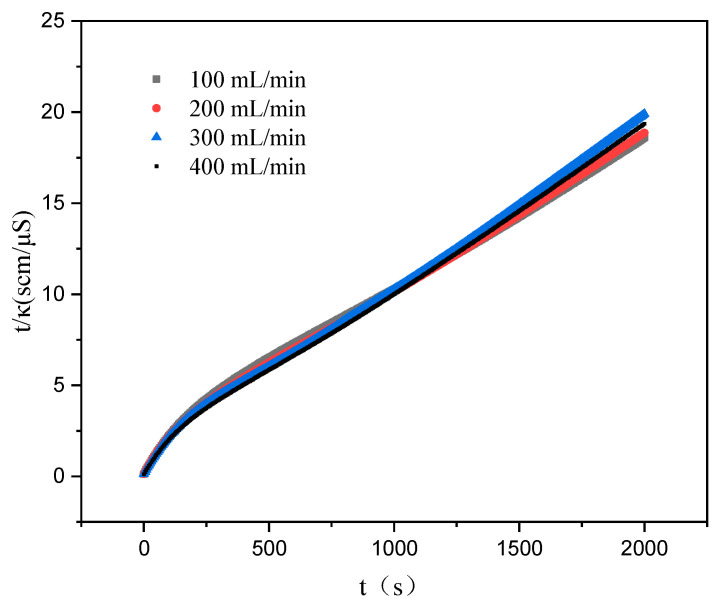
Kinetic fitting curve of the surfactant formation process after injection of CO_2_ into DMDBA at different speeds.

The kinetic equation was fitted to the change in conductivity over time during the formation of DMDBAH surfactant after the injection of CO_2_ into the tertiary amine DMDBA at different speeds (Figure 11 and Figure 12), and the correlation coefficients were all greater than 0.99 (Table 4). This indicates that the formation process of DMDBAH at different injection speeds followed the second-order kinetic law. The faster the injection speed of CO_2_, the more sufficiently the reactants are mixed, which means a higher chance of contact among the reactants, directly accelerating the reaction rate. The rate constant, *k*, reached its maximum value at an injection speed of 400 mL/min, at which the protonation degree of the tertiary amine was not affected (Figure 11).

## 3. Materials and Methods

### 3.1. Reagents and Instruments

Chemical reagents: Dodecylamine, 1,4-dibromobutane, anhydrous formic acid, formaldehyde, anhydrous ethanol, dichloromethane, and sodium hydroxide were purchased from Shanghai Maclin Biochemical Technology Limited Company and were of analytical purity. The crude oil was Gudao heavy oil with a density of 0.9282 g/mL and viscosity (at 50 °C) of 965 mPa·s. The experimental water was secondary distilled water.

Test instruments: Automatic tensiometer (JK99B), Shanghai Zhongchen Technology Equipment Company, Shanghai, China; contact angle measuring instrument (DSA25), Kruss Corporation, Shanghai, China; conductivity meter (DDSJ-308F), Shanghai Yidian Scientific Instrument Company, Shanghai, China; Zeta PALS (NanoBrook 90Plus Zeta), Brookhaven Instruments, Shanghai, China; Fourier transform infrared spectrometer (Spectrum 3), Platinum Elmer Enterprise Management Limited, Shanghai, China; nuclear magnetic resonance spectrometer (Bruker Advance III 400 MHz), Bruker Technology Company, Fällanden, Switzerland; electrospray mass spectrometer (maxis plus), Bruker Technology Company, Beijing, China.

### 3.2. Synthesis

**Scheme 1:**
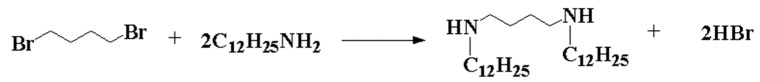
Synthesis of *N*,*N*′-didodecyl butylene diamine.

**Scheme 2:**
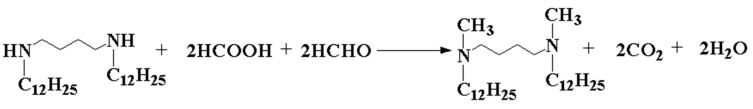
Synthesis of *N*,*N*′-dimethyl-*N*,*N*′-didodecyl butylene diamine.

(1) Dodecylamine and 1,4-dibromobutane were added in a substance ratio of 2.5:1 into a three-mouth flask containing ethanol as the solvent, then the mixture was stirred and refluxed for several hours by heating. The reaction equation is shown in Scheme 1. When the reaction is completed, the reaction mixture was cooled to room temperature, and then filtered to obtain the crude product. Afterward, the crude product was washed with ethanol and deionized water multiple times, and then dissolved into an appropriate amount of dichloromethane; when it was dissolved, 20 wt% NaOH solution was added, and then the mixture was transferred into a liquid separation funnel and left for separation, and the lower layer was collected and evaporated in a rotary evaporator before drying in a vacuum. The yield of the first step is 40~50%.
(2) The product of the first step, formic acid and formaldehyde were added in a substance ratio of 1:6:3 into a three-neck flask containing ethanol as the solvent. Then, the mixture was stirred and refluxed for several hours with heating. The reaction equation is shown in Scheme 2. When the reaction was completed, the reaction mixture was cooled to room temperature, and a 20 wt% NaOH solution was added for pH adjustment to pH = 10–12. Then, the mixture was transferred to the separation funnel and left for stratification. The upper layer was collected and evaporated in a rotary evaporator to yield the final product. The second step’s yield is more than 90%.

### 3.3. Performance Test

#### 3.3.1. Switching Performance

CO_2_ and N_2_ were injected into the DMDBA aqueous solution (gas injection speed 200 mL/min, 25 °C, solution concentration 0.6 wt%), and the changes in the conductivity and Zeta potential of the solution were recorded.

#### 3.3.2. Surface Properties

The surface tension of DMDBAH aqueous solutions at different concentrations was measured by the Du Noüy ring method, by which *γ*/lgc curves were plotted for calculating the critical micelle concentration (CMC), and the surface saturation adsorption capacity (*Γ_max_*), limiting molecular cross-sectional area (*A_min_*) and surface adsorption efficiency (*pC_20_*) were obtained using the Gibbs adsorption formula [36,37,38].

(1) $$\Gamma_{max}=-\frac{1}{2.303nRT}\times {\left(\frac{d\Upsilon }{dlgc}\right)}_{T}$$

(2) $$A_{min}=\frac{1}{N_{A}\Gamma_{max}}$$

(3) $${pC}_{20}=\frac{\Upsilon_{0}-20-\Upsilon_{cmc}}{2.303nRT\Gamma_{max}}-lgcmc$$

where *γ*—surface tension; *lgc*—logarithmic concentration; *dγ*/*dlgc*—slope of the *γ*-*lgc* straight line; *n*—2; *T*—298.15 K; *R*—8.314 J·mol^−1^·K^−1^; *γ*_0_—72.14 mN/m; N*_A_*—6.022 × 10^23^ mol^−1^.

#### 3.3.3. Wettability

The contact angle of the DMDBAH surfactant solution at different concentrations on paraffin film was measured at 25 °C using a contact angle measuring instrument.

#### 3.3.4. Emulsification/Demulsification Performance

The emulsification performance was tested by the water-separating time method. In a test tube with a stopper, 10 wt% model oil (a mixture of oil from Gudao oil field and toluene (1:9, *v*:*v*)) and DMDBAH aqueous solution were added in a ratio of 1:1, and then the volume of oil phase precipitated was observed at different time points after shaking alternately 100 times with right and left hands; the amount of water produced by injecting N_2_ into the emulsion was recorded.

### 3.4. Reaction Kinetics

While injecting CO_2_ into the DMDBA aqueous solution at varying temperatures and injection speeds, the conductivity value of the solution was recorded every 5 s.

The integral method was adopted to find the reaction kinetics by substituting the experimental data into the kinetic equations for different types of reaction orders, and the results indicated that the reaction satisfied the characteristics of the second-order reaction, thereby establishing the following kinetic equation:

(4) $$\frac{d\kappa }{dt}=k{\left(\kappa_{0}-\kappa \right)}^{2}\;$$

where *κ*_0_ is the maximum conductivity, µs·cm^−1^; *κ* is the conductivity value at time *t*, µs·cm^−1^; *t* is the reaction time, s; *k* is the rate constant, μs·(cm·s)^−1^;

When *t* = 0, *κ* = 0; when *t* = *t*, *κ* = *κ*;

Equation (5) is obtained by integrating and sorting Equation (4).

(5) $$\frac{t}{\kappa }=\frac{1}{k\kappa_{0}^{2}}+\frac{t}{\kappa_{0}}$$

From Equation (5), it can be seen that by plotting *t*/*κ* against *t*, the rate constant *k* can be calculated based on the slope and intercept of the line.

## 4. Conclusions

(1) A kind of CO_2_-switchable Gemini surfactant, *N*,*N*′-dimethyl-*N*,*N*′-didodecyl butylene diamine (DMDBA), was synthesized, which enriched the types of switchable Gemini surfactants. Compared with the single-chain surfactant, the CMC of DMDBA surfactant is 1.45 × 10^−4^ mol/L, which is an order of magnitude lower than that of ordinary surfactants, and the surface tension at the CMC (*γ_CMC_*) is 33.4 mN·m^−1^, indicating a more excellent surface activity of the surfactant. Furthermore, the surfactant takes into account the dual effects of emulsion stabilization and simple demulsification. The experimental results show that the introduction of the switchable surfactant during the preparation of an emulsion can save resources and reduce costs. Therefore, the DMDBA surfactant is expected to have promising applications in oil–water separation and other fields.
(2) The process of generating the surfactant by injecting CO_2_ into tertiary amine follows a second-order kinetic equation, with a correlation coefficient greater than 0.9950. It was found that the reaction rate increased with an increase in the injection speed of CO_2_; an appropriate increase in temperature was also conducive to accelerating the reaction, although it reduced the degree of protonation; the activation energy was *Ea* = 91.16 kJ/mol during the formation of the surfactant. This paper provides a theoretical basis for an in-depth understanding of the action mechanism and subsequent applications of switchable tertiary amine surfactants.

## Figures and Tables

**Table 1:** Physical and chemical parameters of surfactants.

Surfactant	CMC/ mol/L	*γ_CMC_*/ mN·m^−1^	*Γ_max_*/ μmol·m^−2^	*A_min_*/ nm^2^	*p*C_20_
Dodecyl dimethyl tertiary amine bicarbonate (C_12_A)	7.41 × 10^−3^	35.6	1.34	1.24	3.21
*N*,*N*′-dimethyl-*N*,*N*′-didodecyl butylene diamine bicarbonate (DMDBAH)	1.45 × 10^−4^	33.4	1.49	1.11	4.94

**Table 2:** The volume of the oil phase precipitated from the emulsion formed by DMDBAH with different concentrations and model oils at different times.

	Concentration	0.1 wt%	0.2 wt%	0.4 wt%	0.6 wt%
Time					
10 min		1.0 mL	0.5 mL	0.0 mL	0.0 mL
30 min		2.0 mL	1.0 mL	0.7 mL	0.7 mL
1 h		4.0 mL	2.0 mL	1.8 mL	1.5 mL
2 h		6.0 mL	3.0 mL	2.8 mL	2.5 mL
3 h		8.0 mL	4.5 mL	3.5 mL	3.0 mL
24 h		14.0 mL	13.0 mL	10.5 mL	10.0 mL

Note: The emulsion is prepared with an oil–water ratio of 1:1 (15 mL:15 mL).

**Table 3:** Kinetic fitting results of the surfactant formation process after injection of CO_2_ into DMDBA at different temperatures.

Temperature /°C	Second-Order Reaction			*k*	Fitting Value of *κ*_0_	Experiment Value of *κ*_0_	Error of *κ*_0_
	Slope	Intercept	R^2^	/(μS·cm^−1^·s^−1^)	/(μS·cm^−1^)	/(μS·cm^−1^)	%
25	0.0085	0.8650	0.9959	8.35 × 10^−5^	117.6	106.4	9.5
35	0.0116	0.6285	0.9951	2.14 × 10^−4^	86.2	79.6	7.6
45	0.0156	0.5092	0.9982	4.78 × 10^−4^	64.1	61.1	3
55	0.0194	0.2002	0.9998	1.88 × 10^−3^	51.54	50.3	2.4

**Table 4:** Kinetic fitting results of the surfactant formation process after injection of CO_2_ into DMDBA at different speeds.

Gas Velocity /(mL/min)	Second-Order Reaction			*k*	Fitting Value of *κ*_0_	Experiment Value of *κ*_0_	Error of *κ*_0_
	Slope	Intercept	R^2^	/(μS·cm^−1^·s^−1^)	/(μS·cm^−1^)	/(μS·cm^−1^)	%
100	0.0083	1.9022	0.9950	3.62 × 10^−5^	120.5	107.8	10.5
200	0.0085	1.6117	0.9970	4.49 × 10^−5^	117.6	106.3	9.6
300	0.0091	1.2484	0.9979	6.63 × 10^−5^	109.9	100.8	8.2
400	0.0090	1.1357	0.9984	7.13 × 10^−5^	111.1	103.6	6.8

## Data Availability

The original contributions presented in the study are included in the article; further inquiries can be directed to the corresponding author.
